# A Pectate Lyase-Coding Gene Abundantly Expressed during Early Stages of Infection Is Required for Full Virulence in *Alternaria brassicicola*

**DOI:** 10.1371/journal.pone.0127140

**Published:** 2015-05-21

**Authors:** Yangrae Cho, Mina Jang, Akhil Srivastava, Jae-Hyuk Jang, Nak-Kyun Soung, Sung-Kyun Ko, Dae-Ook Kang, Jong Seog Ahn, Bo Yeon Kim

**Affiliations:** 1 Incurable Diseases Research Center (WCI), Bio-Therapeutics Research Institute, Korea Research Institute of Bioscience and Biotechnology, Ochang, Chungbuk, 363-883, Republic of Korea; 2 Chemical Biology Research Center, Bio-Therapeutics Research Institute, Korea Research Institute of Bioscience and Biotechnology, Ochang, Chungbuk, 363-883, Republic of Korea; 3 Department of Bio Health Science, Changwon National University, Uichang-gu, Changwon-si, Gyeongsangnam-do, 641-773, Republic of Korea; 4 Stephenson Cancer Center, University of Oklahoma Health Science Center, Oklahoma City, OK, 73104, United States of America; University of Nebraska-Lincoln, UNITED STATES

## Abstract

*Alternaria brassicicola* causes black spot disease of *Brassica* species. The functional importance of pectin digestion enzymes and unidentified phytotoxins in fungal pathogenesis has been suspected but not verified in *A*. *brassicicola*. The fungal transcription factor *AbPf2* is essential for pathogenicity and induces 106 genes during early pathogenesis, including the pectate lyase-coding gene, *PL1332*. The aim of this study was to test the importance and roles of *PL1332* in pathogenesis. We generated deletion strains of the *PL1332* gene, produced heterologous PL1332 proteins, and evaluated their association with virulence. Deletion strains of the *PL1332* gene were approximately 30% less virulent than wild-type *A*. *brassicicola*, without showing differences in colony expansion on solid media and mycelial growth in nutrient-rich liquid media or minimal media with pectins as a major carbon source. Heterologous PL1332 expressed as fusion proteins digested polygalacturons *in vitro*. When the fusion proteins were injected into the apoplast between leaf veins of host plants the tissues turned dark brown and soft, resembling necrotic leaf tissue. The *PL1332* gene was the first example identified as a general toxin-coding gene and virulence factor among the 106 genes regulated by the transcription factor, *AbPf2*. It was also the first gene to have its functions investigated among the 19 pectate lyase genes and several hundred putative cell-wall degrading enzymes in *A*. *brassicicola*. These results further support the importance of the *AbPf2* gene as a key pathogenesis regulator and possible target for agrochemical development.

## Introduction

*Alternaria brassicicola* is a destructive plant pathogen and causes black spot disease on almost all plant species in the Brassicaceae [1–3]. Disease symptoms appear mainly on the leaves and stems of host plants, including *Brassica oleracea* (vegetables), *B*. *rapa* (vegetables, oilseeds, and forages), *B*. *juncea* (vegetables and seed mustard), the vegetable oil-producing species *B*. *napus* (oilseeds) [4], and the model plant *Arabidopsis thaliana* [5]. This disease is of worldwide economic importance [1–3,6,7] and can result in 20 to 50% yield reductions in crops such as canola and rape [7].

*Alternaria brassicicola* is a necrotrophic plant pathogen and its disease symptoms include the necrosis of host tissues, occasionally surrounded by yellow halos. The pathogenesis mechanisms employed by necrotrophic fungi are simplistically described as being comprised of two steps. The first step is the killing of host cells or inducing programmed cell death with toxins [8–14]. The next step is deconstruction of the dead tissue and assimilating it into the fungal biomass using various carbohydrate-active enzymes (CAZys) commonly known as cell wall-degrading enzymes (CWDEs). It has been suspected that toxins and CAZys play important roles in pathogenesis [15], however, we are still searching for genes whose loss-of-function mutation causes a reduction in virulence.

The importance of toxins in pathogenesis has been demonstrated for several necrotrophic fungi [16–18]. Many *A*. *alternata* pathotypes produce secondary metabolites that are host-specific toxins and pathogenicity factors [17,19–25]. Unlike the many pathotypes of *A*. *alternata*, however, no potent toxins associated with pathogenesis have been identified in the brassicaceous pathogen, *A*. *brassicicola*. Only depudecin has been identified as a toxin and its deletion mutants had just a 10% reduction in virulence [26]. Three other toxin candidates, brassicenes [27], brassicicolin A [28], and a protein toxin [29,30], have been discovered, but their association with pathogenesis needs to be characterized by targeted gene mutagenesis. Currently, the evidence of host-specific toxins as pathogenicity factors, or potent general toxins as virulence factors remains tenuous [31,32].

For a successful parasitic lifestyle, efficacious invasion and subsequent colonization are crucial and the number of genes in each family involved in this process are speculated to be increased. Pectin-digesting enzymes are prominent examples. There are 19 pectate lyases and 7 pectin esterases in *A*. *brassicicola*, twice as many as in their homologs in other dothideomycete fungi [33]. Pectin-digesting enzymes are speculated to be involved in the invasion and colonization of host tissues by depolymerizing pectins in the middle lamella and plant cell walls, making them important virulence factors. Six pectate lyase genes (AB05514.1, AB00904.1, AB10322, AB06838.1, AB03608, AB10575.1) are induced by *AbVf19* during the late stages of infection, after establishment and colonization, when plant tissues are necrotic [34]. Loss-of-function mutations of the most abundantly expressed gene (AB10322.1) or other pectate lyase genes, however, do not result in a reduction in virulence [15]. This suggests that the lost function of individual pectin digestion enzymes is either replaced or complemented by unknown enzymes. Alternatively, the major function of AB10322.1 is in something other than pathogenesis. Functional redundancy among CAZys, and functional specialization of individual genes within each family have been proposed previously to explain similar observations in *Cochliobolus carbonum* [35].

Recently, we identified two pectate lyase genes, *PL1332* (AB01332.1) and *PL4813* (AB04813.1), which are exponentially induced as early as 4 hours after fungal contact with the surface of its host and lasting up to 24 hours postinoculation [36]. These two genes are regulated by the transcription factor *AbPf2*, which is involved in the early stage of pathogenesis. Deletion strains of the *AbPf2* gene are nonpathogenic, but its other phenotypes are the same as wild-type *A*. *brassicicola* in saprophytic growth, both in the presence and absence of stress-inducing chemicals [36]. In this study, we tested a hypothesis that the *PL1332* gene encoding a pectin digestion enzyme is an important virulence factor. The results of this study provide another reason to further investigate the functions of other genes regulated by *AbPf2* and to consider this transcription factor a good target for efficient management of diseases caused by *A*. *brassicicola*.

## Results

### Expression patterns of pectate lyase genes during plant infection and saprophytic growth

**Fig 1:**
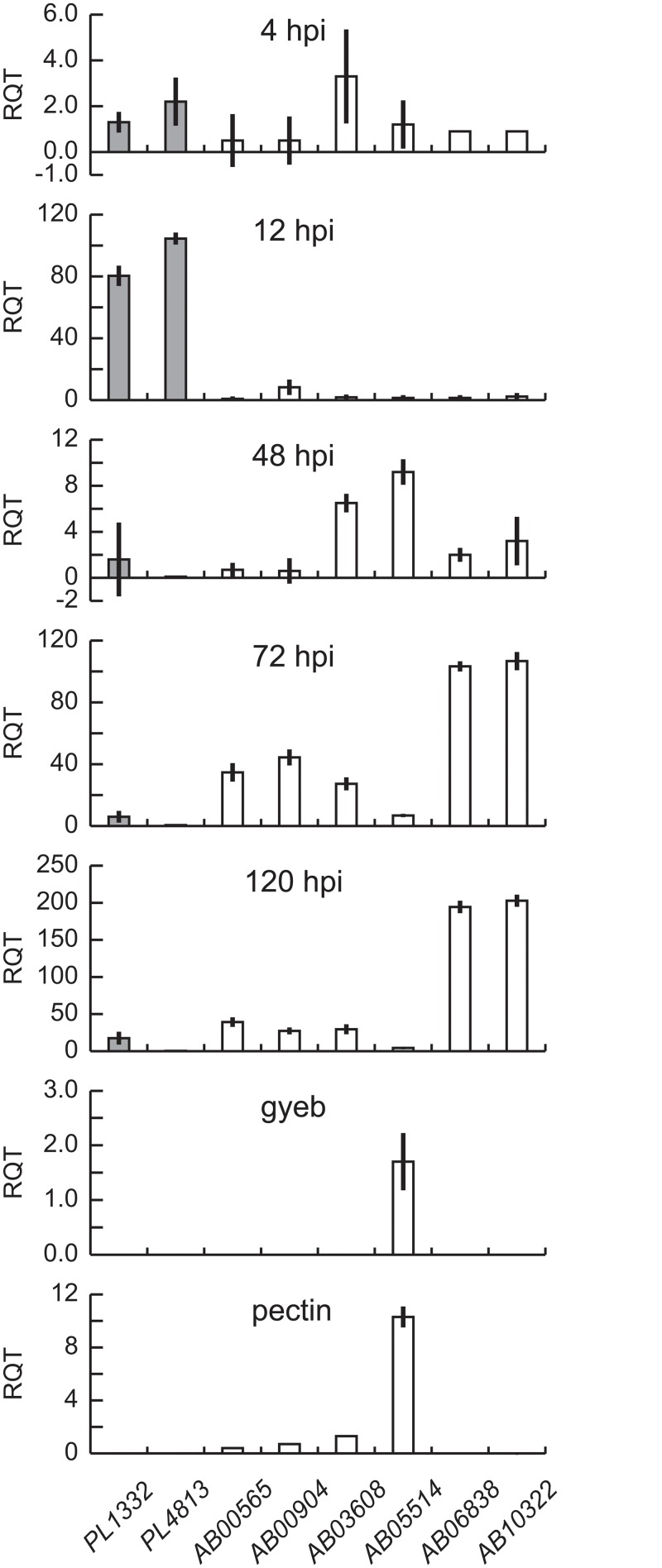
Differential expression of eight pectate lyase-coding genes. Relative amount of transcripts for eight individual pectate lyase-coding genes in wild-type *Alternaria brassicicola*. The amount of transcripts for each gene is shown as a percentage of the amounts of *Ef1-α* transcripts at each stage. Y-axes indicate the relative quantity of the transcripts (RQT) of each gene compared to *Ef1- α*. Gene names are shown below the X-axes. Error bars indicate standard deviation (N = 3). hpi: hours postinoculation, when the fungal tissues were harvested; gyeb: fungal mycelium grown in glucose yeast extract broth; pectin: fungal mycelium grown in a minimal medium supplemented with pectin as a major carbon source.

Two putative pectate lyase-coding genes, *PL1332* and *PL4813*, regulated by the transcription factor *AbPf2*, were dramatically induced soon after conidia were inoculated on leaves of host plants [36]. Expression levels of these genes and six other pectate lyase genes were further quantified and compared with transcripts of a gene encoding elongation factor 1-α (*Ef1-α*) (Fig 1). The expression levels of *Ef1-α* were more consistent than all other genes encoding housekeeping proteins [36]. The expression levels of all eight pectate lyase genes were less than 3% of the transcripts of *Ef1-α* at 4 hours postinoculation (hpi) (Fig 1A), but transcript levels of *PL1332* and *PL4831* were dramatically increased afterwards and reached levels comparable to *Ef1-α* by 12 hpi (Fig 1B). Subsequently, their expression levels decreased to less than 2% by 48 hpi when colonization was established. The expression levels of these genes remained low during saprophytic growth on both dead host tissue and axenic media (Fig 1D–1G). Notably, the presence of pectin as a major carbon source did not induce their expression (Fig 1G). The other six pectate lyase-coding genes (AB05514.1, AB00904.1, AB10322, AB06838.1, AB03608, AB10575.1) are putatively regulated by the *AbVf19* transcription factor [34]. Although their expression was induced by *AbVf19* during the late stage of infection, the magnitude of induction varied from less than 5% to over 200% compared to the expression levels of *Ef1-α*. Particularly, AB10322 and AB06838 among the six pectate lyase-coding genes were expressed at their highest levels during the late stages of infection. All eight of the pectate lyase-coding genes were expressed at low levels during saprophytic growth in a liquid culture medium and none were induced when pectin was a major carbon source in the medium.

### Sequence similarity between two genes encoding pectate lyases

**Fig 2:**
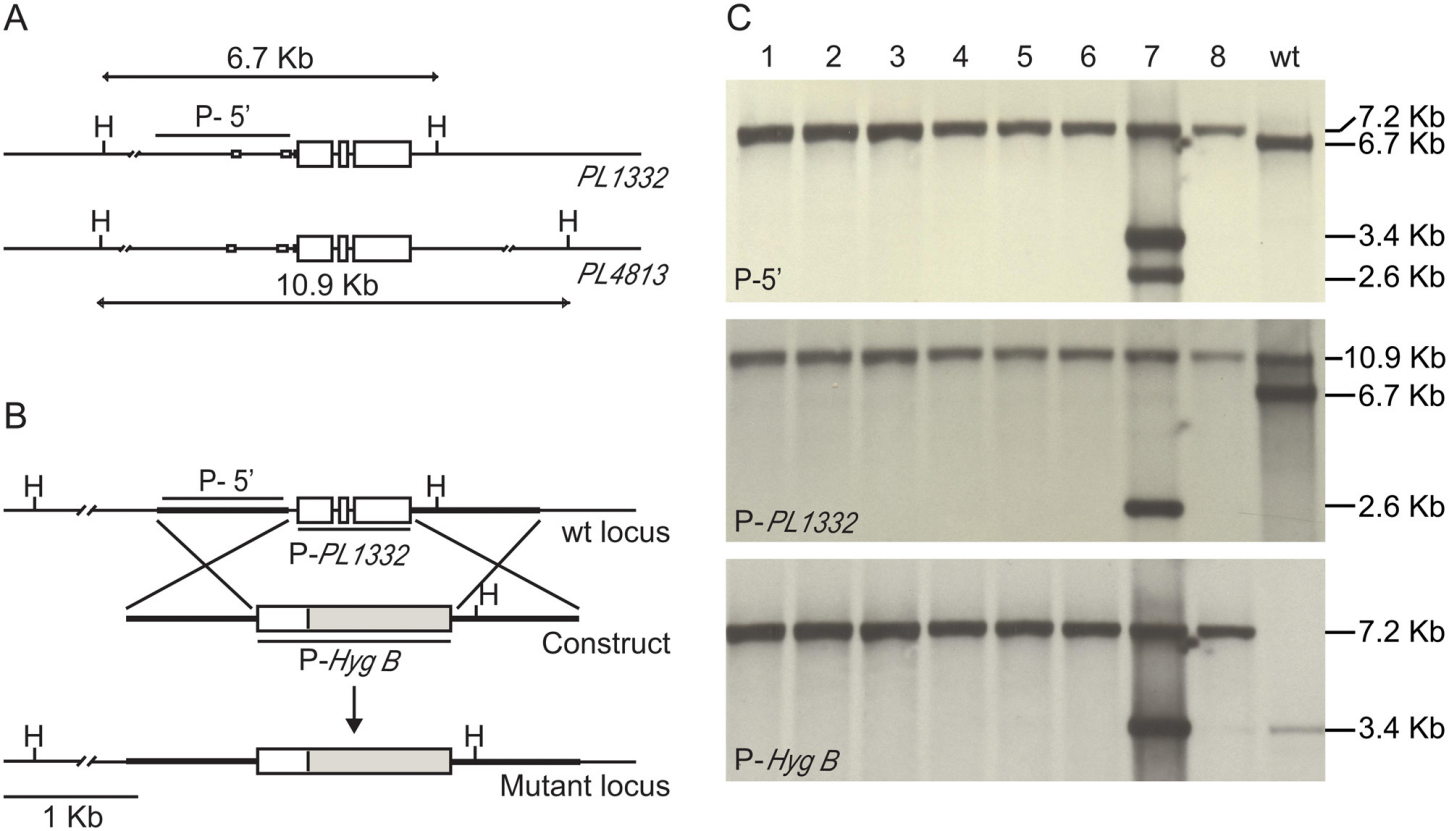
Selective deletion of the *PL1332* gene without affecting the *PL4813* gene. A. Schematic diagram of sequence comparisons between *PL1332* and *PL4813* loci. Three filled boxes at the 5’ side show blocks of similar sequences between the two genes. B. Schematic diagram of the wild-type locus, exogenus construct, and mutant locus. The mutant locus represents replacement of the *PL1332* coding region with a single copy of a selectable marker, Hygromycin B (*HygB*) resistance cassette. C. Southern blots. The top panel shows a band shift from 6.7 Kb to 7.2 Kb by replacing 915 base pairs of the *PL1332* coding and flanking region with 1436 base pairs of *HygB* cassette. The middle panel shows the PL4832 gene represented by a 10.9 kb band in all isolates, while the absence of *PL1332* is represented by a 6.7 Kb band in all mutants compared to the wild type. This band does not appear in the top panel because sequence similarity was low at the probing region. The bottom panel shows the *HygB* cassette in all mutants except the wild type. Mutants represented by DNA lanes 1 and 2 were used for pathogenesis assays. The Δ*pl1332-1* mutant was complemented with a wild-type allele and mainly used for the virulence assays. P5’, P-P*L1332*, and P-*Hyg* indicate locations of the Southern probes. H indicates *Hin*dIII enzyme digestion sites.

In addition to their similar expression pattern, the *PL1332* and *PL4813* genes shared three short blocks of similar sequences within a 1 kb sequence upstream from the start codon (Fig 2A) and an identical motif of a putative promoter [36]. The length of its genomic DNA was 835 nucleotides and contained one putative intron and two exons. We determined their cDNA sequence and defined the coding region for both genes (Fig 2A). There were three exons and two introns in each gene. The length of the coding regions and their nucleotide sequences were similar (Table 1 and S1 Fig). Their introns were also similar in their location, length, and sequence. Similarity in their expression profiles, gene structures, and gene sequences suggested that their functions were similar. For this reason, we decided to study one of the two genes instead of both.

**Table 1:** Comparisons of sequence similarity between *PL1332* and *PL4813*.

	P1	P2	P3	E1	I1	E2	I2	E3	Coding region	Intron combined
PL1332	73 nt	86 nt	32 nt	253 nt	56 nt	60 nt	50 nt	416 nt	729 nt	106 nt
PL4813	73 nt	89 nt	36 nt	253 nt	51 nt	60 nt	50 nt	419 nt	732 nt	101 nt
Alignment	59/73	69/89	31/36	200/256	28/56	54/60	33/50	285/350	544/671	61/106
% identity	81%	78%	86%	78%	50%	90%	66%	81%	81%	58%

P: sequence blocks at 5’ upstream sequence from the start codon, E, I: an exon and an intron.

### Replacement of *PL1332* with a HygB cassette

We designed a construct to create deletion strains of the *PL1332* gene by replacing the coding region with a Hygromycin B transferase (*HygB*) gene cassette (Fig 2B). Southern hybridization with three probes against the genomic DNA extracted from eight transformants confirmed that the *PL1332* gene was absent in all eight transformants (Fig 2C). The *PL1332* coding region was replaced by a single copy of the *HygB* resistance cassette in seven strains and by multiple copies in one of the gene-deletion strains (*Δpl1332-7*). In contrast to replacement of the *PL1332* gene with a *HygB* cassette, the *PL4813* gene was left intact in all strains.

### Reduction in virulence of the Δ*pl1332* strains

**Fig 3:**
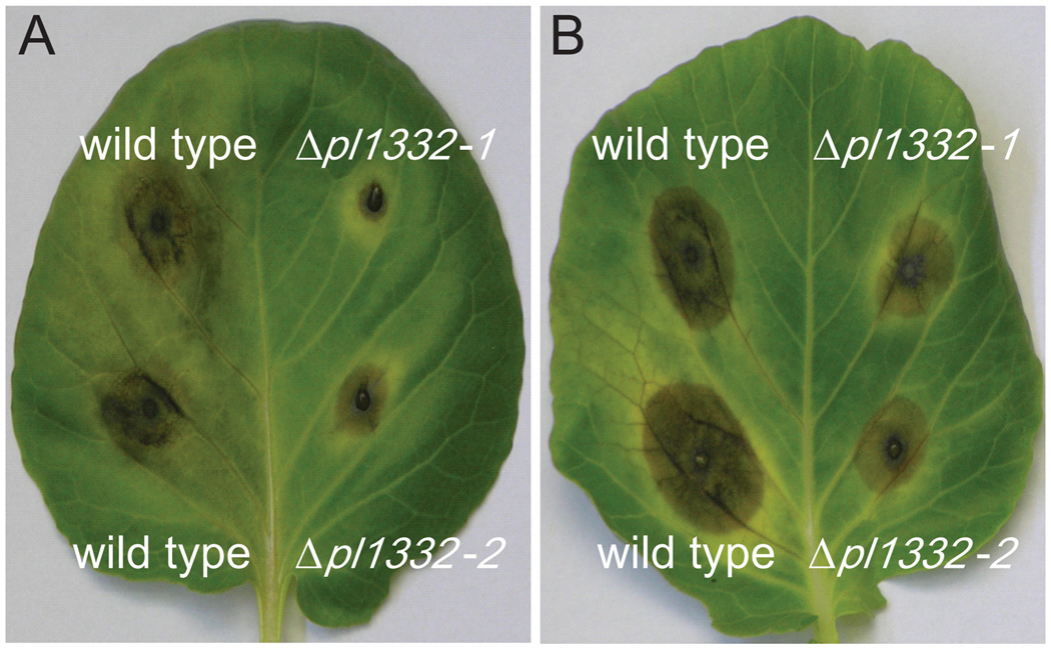
Reduced virulence of mutants on *Brassica oleracea*. Lesions caused by two strains of *Δpl1332* mutants and wild-type *A*. *brassicicola* on leaves of green cabbage, *B*. *oleracea*. Pictures were taken 5 days postinoculation. A. Assay results on a detached leaf. B. Assay results on leaves attached to plants (leaves detached for photographing).

We performed virulence assays using two strains, Δ*pl1332-1* and Δ*pl1332-2*, to further characterize virulence attributes associated with *PL1332*. Both deletion strains produced lesions approximately 30% smaller in diameter (*p*<0.001) than the wild type in detached-leaf assays (Table 2 and Fig 3A). We performed similar experiments using the Δ*pl1332-1* strain on leaves still attached to the plant. The size of lesions produced by **Δ** *pl1332-1* strain was similarly reduced compared to the wild type on the leaves attached to whole plants (Fig 3B). We also compared lesions produced by the wild-type, ***Δ*** *pl1332-1* strain, and a strain complemented with the wild-type allele. Lesions produced by the complemented strain were similar to those produced by the wild type (Table 2). Results of these virulence assays provided evidence that loss of the *PL1332* gene caused the reduction in virulence and that *PL1332* was important for full virulence.

**Table 2:** Decreased virulence of *PL1332* deletion mutants compared with wild-type *Alternaria brassicicola*.

Mutant	Degrees of freedom	Leaf type	Wild type (mm)^1^	Mutant (mm)^1^	Virulence (% decrease)	Probability
Δ*Pl1332-1*	12	Detached leaves	19.3 ± 2.9	11.3 ± 5.7	41.4	1.48E-04
Δ*Pl1332-2*	8	Detached leaves	17.4 ± 2.6	13.3 ± 2.6	23.6	6.70E-04
Δ*Pl1332-1*	12	Attached leaves	16.2 ± 3.4	10.2 ± 4.8	37.4	2.42E-05
Δ*Pl1332-1* complemented	8	Detached leaves	17.5 ± 4.4	16.6 ± 4.1	4.8	0.03

^1^ Lesion size is the average lesion diameter ± standard deviation in millimeter. Probability was calculated by Student *t*-test.

### No differences in vegetative growth

We evaluated the importance of *PL133*2 on the growth of colonies on solid media and on mycelium production in liquid media that were either rich in nutrients, or contained only essential minerals supplemented with pectin as a major carbon source. On solid, nutrient-rich PDA, the average colony diameter was similar for the deletion strain and wild-type *A*. *brassicicola* (Table 3). Colony size was also similar for the Δ*pl1332-1* strain and wild type on minimal mineral agar supplemented with pectin. We inoculated two different liquid media with fungal mycelium from either the Δ*pl1332-1* strain or wild-type *A*. *brassicicola*, and then measured their dry weights four days later. Dry weight of the mycelium was similar for the mutant strain and the wild type in nutrient-rich GYEB as well as in a minimal medium supplemented with citrus pectin or glucose as a major carbon source (Table 4). Both strains and the wild type grew poorly in the minimal media supplemented with citrus pectin or glucose compared to growth in nutrient-rich media. This suggested that pectin digestion enzymes were not induced by citrus pectin and that PL1332 was not important in the use of citrus pectin.

**Table 3:** Growth of wild-type and the Δ*pl1332-1* strain of *Alternaria brassicicola* on PDA or on water agar with 1% (w/v) pectin.

Chemical	Colony diameter (mm)	
	Wild type	Δ*pl1332-1*
MMA + pectin	18 ± 0	18 ± 0
PDA	17.5 ± 0	17.5 ± 0

Colony diameter indicates the average colony diameter ± standard deviation.

**Table 4:** Similar rates of vegetative growth between the Δ*pl1332* mutant and wild-type *Alternaria brassicicola* in the presence of different carbon sources.

Carbon source	Medium type	Degrees of freedom	Wild type (μg)^1^	Δ*pl1332-1* (μg)^1^	Difference (%)	Probability
Pectin	Broth	2	21.0 ± 7.1	21.3 ± 7.4	1.43	0.95
Glucose	Broth	2	20.0 ± 2.8	19.9 ± 2.0	-0.67	0.96
GYEB	Broth	2	127.8 ± 11.8	126.4 ± 18.3	-1.10	0.92

^1^ Dry weight indicates the average mass ± standard deviation.

### Enzyme activities of PL1332 expressed in *Escherichia coli*

**Fig 4:**
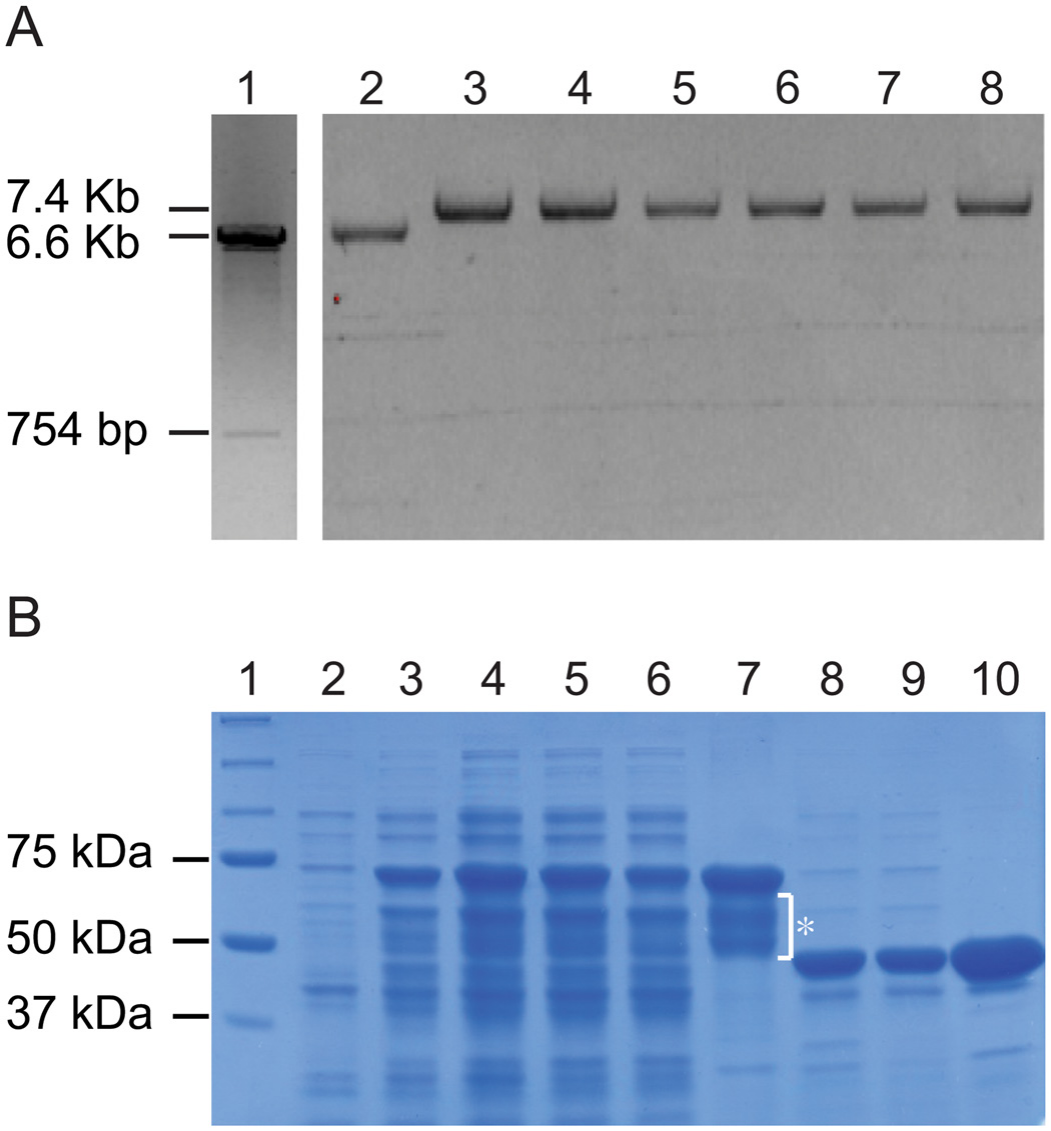
Expression of PL1332 proteins in *Escherichia coli*. A. Cloning of *PL1332* cDNA and successful transformation of the expression vector. Lane 1: *PL1332* cDNA pMAL-c2x expression vector after digestion with *Bam*H1 and *Hin*dIII; Lane 2: empty pMAL-c2x vector; Lanes 3–8: recombinant plasmids purified from *E*. *coli* transformed with the expression construct. All plasmids in lanes 2–8 were digested with *Bam*H1 while the plasmid in lane 1 was digested with *Bam*H1 and *Hin*dIII. The clone shown in lane 3 was used for protein production. B. Expression of the maltose binding protein (MBP) and PL1332 fusion (MBP-PL1332) protein. Lane 1: protein markers; Lane 2: total crude extract before IPTG induction; Lane 3: total crude extract after IPTG induction; Lane 4: total crude extract; Lane 5: supernatant of the lysate; Lane 6: flow through; Lane 7: purified protein. Expected size of the MBP-PL1332 in lanes 2 through 7 was 68 KD and smaller bands marked with an asterisk are degraded fusion proteins; Lane 8: total crude extract of MBP protein; Lane 9: supernatant of the lysate; Lane 10: purified MBP. Expected size of MBP protein in lanes 8 through 10 was 42.5 KD.

We failed to measure knockout effects of the *PL1332* gene on the enzyme activity of pectate lyases secreted in the culture medium because the *PL1332* gene was expressed at extremely low levels in the liquid medium, with or without pectin (Fig 1, GYEB and pectin). Further, it was not possible to measure enzyme activity in the inoculum collected from the infection sites when the *PL1332* gene was highly induced because the fungal biomass was extremely small at 4 to 24 hours postinoculation. To verify its enzyme activity, we expressed the PL1332 protein by cloning the gene in a heterologous protein expression system (Fig 4A). PL1332 was expressed as a fusion protein by linking it to maltose binding protein (MBP) (Fig 4B). Two amino acids at the N-terminus were deleted during the cloning of *PL1332* cDNA in the proper reading frame, following the MBP-coding region. After IPTG-was induced, all transformants abundantly expressed the ~68 KDa proteins expected from the fusion of MBP and PL1332 (MBP-PL1332). The fusion proteins were soluble and stayed in the cytosol of *E*. *coli* cells. However, the proteins were partially degraded after purification, unlike the intact MBP proteins (Fig 4B, compare lane 7 and lane 10). Treatment of the fusion protein with Factor Xa to remove the MBP domain caused complete degradation of the protein during overnight incubation at 4°C (data not shown). Thus, we were not able to perform enzyme assays using PL1332 after removing the MBP binding domain. Instead we performed enzymatic assays using the fusion proteins that were partially degraded. Enzyme activity was measured by the extent of enzymatic digestion of polygalacturonic acid to oligogalacturonic acid using a titrimetric stop-reaction method.

**Fig 5:**
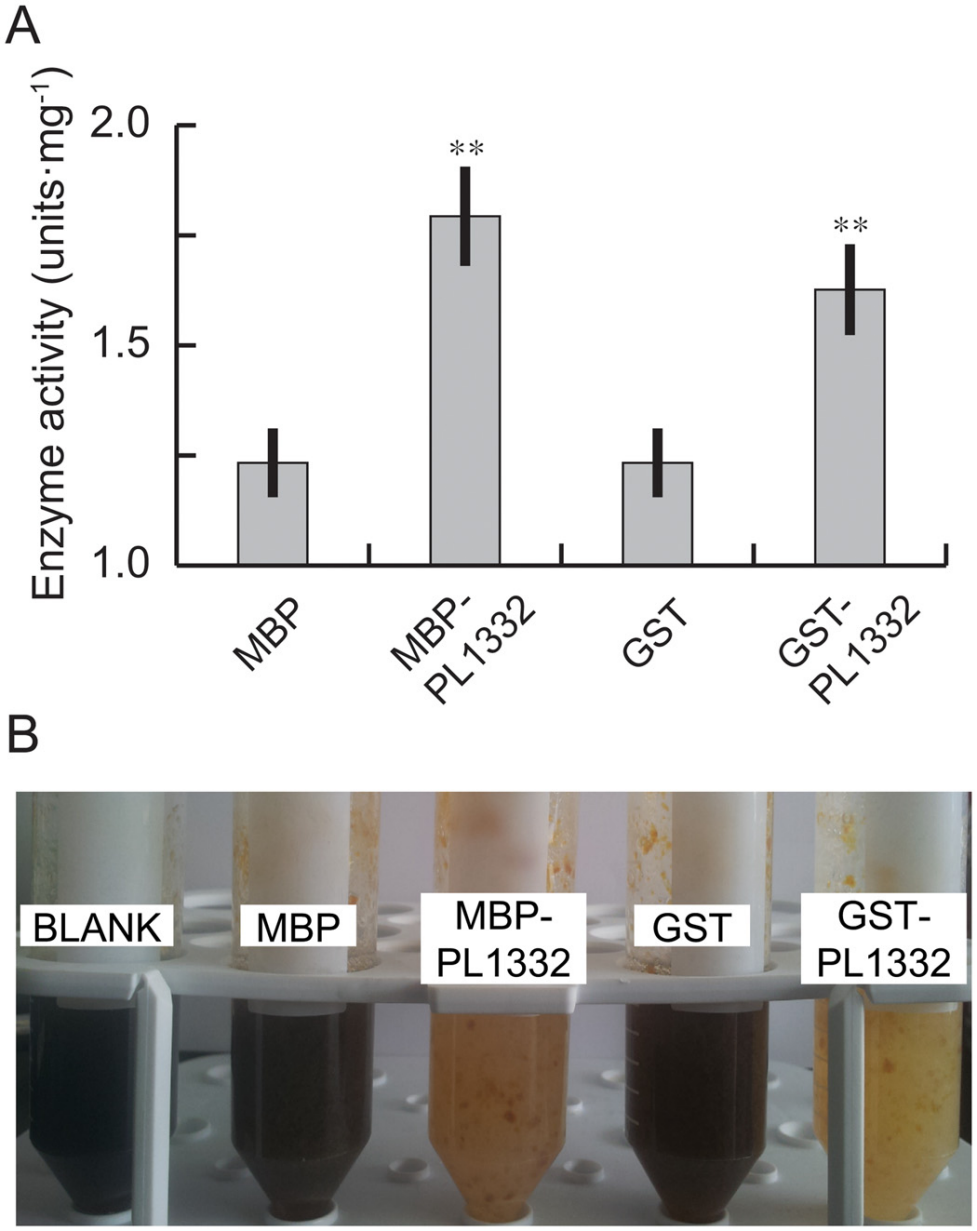
Enzyme activity of pectate lyase measured by a titrimetric stop reaction method. A. Enzyme activity calculated by free iodines not covalently bound to oligogalacturonic acids that originated in polygalacturonic acids. MBP: maltose binding protein, MBP-PL1332: fusion protein, GST: glutathione-S-transferase, GST-PL1332: fusion protein. B. Visual comparison of the relative amounts of free iodine. Intensity of dark brown color indicates relative amounts of free iodine, which inversely correlates with enzyme activity. ** *p* < 0.01.

Purified MBP-PL1332 fusion proteins did not show enzyme activity under any of the test conditions (S2 Fig). We speculated that the fusion proteins were either degraded during protein purification and subsequent enzyme-assay conditions, or the enzyme required unknown co-factors for its activity. To circumvent possible problems caused by protein degradation, the absence of unknown cofactors in the reaction mixture, or both, we measured enzyme activity in the soluble fraction of whole lysates of *E*. *coli* that expressed MBP-PL1332 fusion proteins. Soluble lysate of *E*. *coli* expressing MBP was used as a control and it showed weak enzyme activity, as expected (Fig 5 MBP-PL1332). The soluble lysate of *E*. *coli* expressing MBP-PL1332 fusion proteins, however, showed significantly stronger enzyme activity (*p* < 0.01) than the control. We performed similar experiments using PL1332 proteins fused to glutathione-S-transferase (GST). The GST-PL1332 fusion proteins in soluble bacterial lysate also showed stronger enzyme activity than GST in soluble bacterial lysate (Fig 5 GST-PL1332).

### Induced necrosis of host tissue

**Fig 6:**
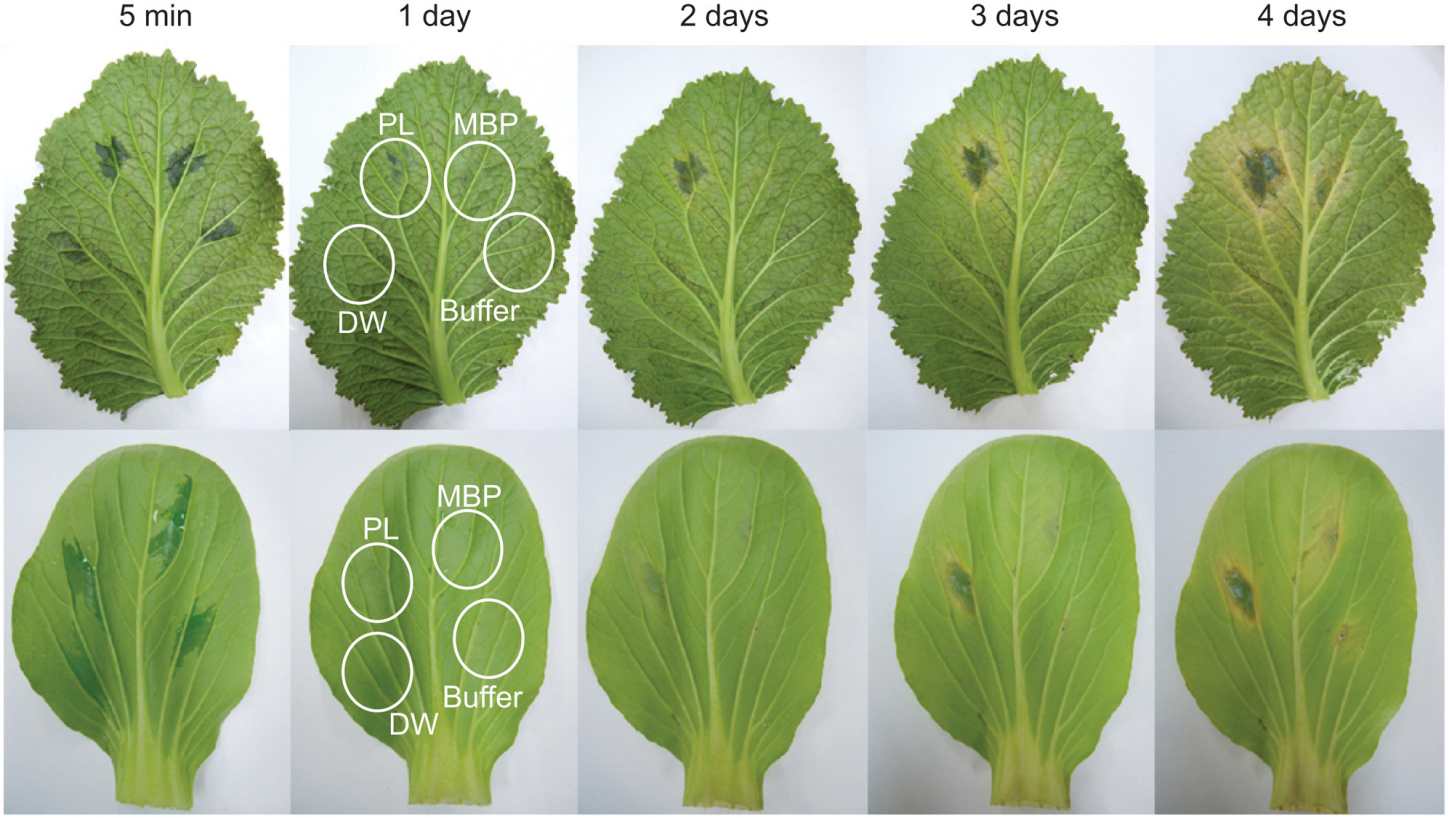
Necrosis of leaf tissue on *Brassica juncea* and *B*. *campestris* caused by bacterial lysates containing MBP-PL1332 fusion proteins. PL: MBP-PL1332 fusion proteins in a soluble fraction of bacterial lysate; MBP: Maltose binding proteins in a soluble fraction of bacterial lysate; Buffer: 10 mM maltose in wash/elution buffer (20 mM Tris-HCl, pH 7.4, 200 mM NaCl, 1 mM EDTA, 10 mM β-mercaptoethanol); DW: deionized water.

Toxins play important roles in necrotrophic parasitism in other fungi and we have been searching for similar toxins in *A*. *brassicicola*. We considered pectate lyases in general as toxin candidates because pectins are important components of the architecture of plant tissue. We tested whether PL1332 protein was toxic to host plants using MBP-PL1332 fusion proteins in a soluble fraction of bacterial lysate. Maltose binding protein in soluble bacterial lysate, protein wash buffer, or sterilized deionized water were used as controls. When each solution was injected between the leaf veins of host plants, local tissues around the injection sites immediately appeared waterlogged, but the symptom disappeared in about 3 hours (Fig 6). The leaf tissue of *B*. *juncea* injected with MBP in soluble bacterial lysate, protein elution buffer, or deionized water remained symptomless for up to 7 days, when the experiment ended. Minor symptoms occasionally appeared at the injection sites of *Brassica campestris var*. *chinensis* as a result of probable secondary infection. In contrast, when MBP-PL1332 in soluble bacterial lysate was injected, necrotic symptoms appeared as early as one day after injection and gradually expanded. We also tested if the purified MBP-PL1332 fusion proteins remained toxic, though the proteins showed no enzyme activity (S2 Fig). Leaf tissue injected with the purified fusion proteins produced necrotic symptoms 2 days postinoculation on both host plants and continued to expand gradually (S3 Fig). In contrast, purified MBP, elution buffer, or water did not cause necrosis in control experiments although multiple black spots and chlorosis on all over the leaves of *B*. *campestris var*. *chinensis* often appeared after injection of the control samples. These spots were thought to be from secondary infections caused by unknown organisms (S3 Fig).

## Discussion

Pectins are structural heteropolysaccharides and key components of primary and secondary cell walls of flowering plants, and they are important for the protection of plants from abiotic stresses and biotic invasion [37–39]. Genes encoding pectinolytic enzymes are important virulence factors and their deletion or disruption causes a reduction in virulence of several phytopathogenic fungi, such as *Aspergillus flavus*, *Botrytis cinerea*, and *Claviceps purpurea* [40–42]. The genome of *A*. *brassicicola* contains about twice the number of genes encoding pectin-digestion enzymes as other dothideomycete fungi [33]. These enzymes are probably important for its pathogenic lifestyle, but evidence has been lacking until now. Previously, the disruption of a pectate lyase gene that was abundantly expressed during plant infection caused little or no reduction in the virulence of *A*. *brassicicola* [15]. Further, disruption of four other putative pectate lyase genes in this necrotroph did not change its virulence (Cho, unpublished data). Identification of individual pectate lyase genes associated with pathogenesis had been challenging until pectate lyase gene *PL1332* was identified on the molecular level as an important virulence factor.

This study clarifies why previous approaches were unsuccessful in identifying pectate lyase genes important in pathogenesis. There are 19 pectate lyase genes and 7 pectin esterase genes in *A*. *brassicicola* [33]. The pectate lyase-coding gene, AB10322, expressed at high levels by the fungal mycelium in necrotic plant tissue during host infection, was considered important for pathogenesis and selected as the best candidate for a virulence factor. In retrospect, though AB10322 was abundantly expressed during the late stages of infection, it was poorly expressed during the early stages of infection. Furthermore, four additional genes were also highly expressed during the late stage of infection (Fig 1). Pectate lyases encoded by AB10322 and the other four genes probably play important roles in deconstructing pectins and unlocking sugars for use as basic structural components of the fungal biomass. These available sugars would be important for subsequent colony expansion of the necrotroph and appearance of the typical disease symptom: macerated plant tissue. Thus, a gene-disruption strain was expected to result in a slower expansion of disease symptoms compared to the wild type, in contrast to no changes by the disruption of AB10322. A major reason for the unchanged virulence of the *ab10322* strain was probably functional redundancy among the five pectate lyase-coding genes expressed at moderate to high levels during the late stage of infection (Fig 1). Functional redundancy was previously proposed for another pathogenic fungus, *Cochliobolus carbonum* [35].

These experimental results offer answers to lingering questions on the role of toxins in the pathogenesis of *A*. *brassicicola*. PL1332 was a strong toxin and caused necrosis in the host plants tested (Fig 6). This protein, however, was smaller in molecular weight than the previously reported 35 KDa AB-toxin [29,30]. It was also different from host-specific toxins that are secondary metabolites produced by several pathotypes of *A*. *alternata* [17,21,22,24,25]. These secondary metabolites are toxic to selected host plants and essential for pathogenicity. In comparison, PL1332 was toxic to all three host plants tested and gene-deletion strains were still pathogenic, suggesting that it is a general toxin rather than a host-specific toxin.

Pectins are major components of plant cell walls and the middle lamellae that help bind cells together. Therefore, the digestion of pectins will cause tissue collapse, cell membrane rupture, and subsequent tissue necrosis. It is also possible that oligopectins, or pectin derivatives digested by the enzyme, triggered host defense reactions [43] and programmed cell death. In these cases, enzyme activity was necessary to cause the necrosis. Alternatively, the enzyme activity of PL1332 is not required for necrosis, nor is the xylanase activity of *Xyn11A* secreted by *B*. *cinerea* [12]. Thirty amino acids in the xylanase *Xyn11A* are sufficient for toxicity, even without enzyme activity of xylanase. The latter idea was supported in our study when purified fusion proteins showed no detectable levels of enzyme activity (S2 Fig), but produced toxic effects (S3 Fig). This observation suggests that pectate-lyase enzyme activity is not necessary for toxicity. Further study is needed to clarify whether the toxic effects of PL1332 on host tissues resulted from or were independent of the enzyme activity. If the toxic effect is not caused by pectate lyase activity, introduction of the Δ*pl1332* with a defective PL1332 enzyme, or expression cassettes for short oligopepetide coded by PL1332, would restore full virulence and defective PL1332 proteins or short oligopeptides would still be toxic to host plants.

Regardless of the molecular mechanism, PL1332 is an important general toxin and it affects more than one species of host plant. It is the first protein molecularly substantiated in *A*. *brassicicola* that shows toxicity to host tissue. Unlike the five pectate lyase genes abundantly expressed during the late stages of infection, the *PL1332* and *PL4813* genes were highly expressed during early infection. These results suggest that these genes specifically interact with host plants early in the infection process, before or during penetration, rather than later during the conversion of sugars and colony expansion. This study raises several interesting questions, including the roles of the *PL4813* gene on virulence, functional redundancy between the *PL1332* and *PL4813* genes, possible synergistic effects of the mutation of both genes, and the importance of the five late-stage genes in virulence.

This study was initiated based on discovery of the key-pathogenesis regulator, *AbPf2* [36]. Its transcription was induced during the early stages of host infection, followed by the induction of 106 putative downstream genes, including *PL1332* [36]. Data generated in this study provide the first evidence that these downstream genes might be important in pathogenesis. It is of note that this transcription factor also regulates six genes that encode small secretion proteins. They may act as effectors and be important in the interaction between host plants and pathogenic fungi [44–46] or fungus-like oomycete pathogens [47,48]. It would be practical and fruitful to explore the roles of these six putative effector genes that are explosively induced during the brief early infection stage. They may increase our understanding of the biological aspect of pathogenesis, especially the mode of secretion of these effector proteins and host—pathogen interactions. Our study results also provide a reason why the deletion or disruption of the *AbPf2* gene causes a loss of pathogenicity in *A*. *brassicicola*. Inhibition of AbPf2 would provide full protection to plants from *A*. *brassicicola* infection, while inhibition of pectae lyase activities would provide partial protection. Therefore, inhibition of *AbPf2* would be a better target than individual downstream genes, including PL1332. It is feasible to screen natural or synthetic compounds that inhibit the functions of *AbPf2* and ultimately pathogenesis.

## Experimental Procedures

### Maintenance of fungal strains and Southern hybridization

Growth and maintenance of *Alternaria brassicicola* Schweinitz & Wiltshire (ATCC96836), pathogenicity assays using deletion strains or wild-type *A*. *brassicicola*, and its transformation and nucleic acid isolation were performed as described previously [49]. Each strain created during this study was purified by two rounds of single-spore isolation to obtain a uniform genetic background. Loss-of-function mutation was verified by Southern hybridization using three probes, respectively representing the 5’ upstream region, PL1332 coding region, or HygB gene cassette. Southern hybridizations were performed as described previously [15] following manufacturer’s protocol (Roche Diagnostics, Mannheim, Germany) with appropriate modifications. Fungal DNA extracted from each transformant or wild-type *A*. *brassicicola* was digested with the endonuclease, *Hin*dIII. All three probes were synthesized with a PCR DIG Probe Synthesis Kit according to the manufacturer’s manual (Roche Diagnostics, Mannheim, Germany). A gene-specific probe was generated with the primer set 1332ProbeF (CCCTCAACATCCCAGCTAGA) and 1332ProbeR (TGTTAATGGCGACAAGGTCA). The probe for the 5’ flanking region was produced with 1332-DP1 (CGCACCCGTAAGAAGAAGAA) and 1332-DP2’ (TTCAAAGTGGCAGAGCACAC), and the *HygB*-specific probe was produced with HygIn84 (CTTGGCTGGAGCTAGTGGAG) and HygIn1343 (ATTTGTGTACGCCCGACAGT). Gene-deletion strains were maintained as glycerol stock in separate tubes with one tube used for each assay. The sequence data were deposited in the NCBI GenBank (KR024320-KR024323).

### Pathogenicity assays

Either whole plants or detached leaves harvested from 5- to 8-week-old *Brassica oleracea* (green cabbage) were inoculated with 1–2 x 10^3^ conidia in 10 μl of water. After infection, the plant materials were maintained in mini humidity chambers and the development of disease symptoms observed for 7 days. Pathogenicity assays were conducted multiple times and the disease symptoms recorded 5 dpi with a digital camera.

### Creation of gene deletion-strains for *PL1332*

We made Δ*pl1332* deletion strains by replacing the 915 base pairs (bp) spanning the partial promoter (70 bp), whole protein-coding region (835 bp), and partial sequence of 3’ untranslated region (bp nt) with a *HygB* resistance cassette (Fig 2B). The replacement construct was produced by two rounds of PCR as described previously [36]. Initially, a 978-bp-long 5’ flanking region of the PL1332 gene, 1436-bp-long HygB cassette, and 954-bp-long 3’ flanking region were amplified with three sets of primers; 1332-DP1 and 1332-DP2 (ATCAGTTAACGTCGACCTCGTTCAAAGTGGCAGAGCACAC); 1332-DP3 (GTGTGCTCTGCCACTTTGAACGAGGTCGACGTTAACTGAT and1332-DP4 (ATTGTGCTTTCCGTGGAGTCCGTCGACGTTAACTGGTTCC); 1332-DP5 (GGAACCAGTTAACGTCGACGGACTCCACGGAAAGCACAAT) and 1332-DP6 (AACTTTTCGGCAAAATCTCG). Subsequently, PCR products were mixed and used as template DNA to create the final construct by amplifying DNA with 1332-DP1 and 1332-DP6. Sequence similarity between PL1332 and PL4813 at the 5’ flanking region was undetectable except for three short blocks marked in Fig 2A. In addition, there was no sequence similarity at the 3’ flanking region. In short, the construct was designed to replace only the PL1332 coding region without affecting the PL4813 coding region. The final construct was transformed into the protoplast of wild-type *A*. *brassicicola* as described previously [15,36].

### Complementation of Δ*abpf2-1* strain

The Δ*Pl1332-1* strain was complemented with the wild-type *PL1332* allele and its native promoter as described previously [50].

### Growth assays in the presence of major carbon sources

Colony growth assays were performed on either potato dextrose agar (PDA) plates or water agar plates. Water agar (2% w/v) plates contained 0.5% (NH_4_)_2_SO_4_, 0.15% KH_2_PO_4_, 0.06% MgSO_4_, 0.06% CaCl_2_, 0.0005% FeSO_4_ 7H_2_O, 0.00016% MnSO_4_ H_2_O, 0.00014% ZnSO_4_ 7H_2_O, and 0.00037% CoCl_2_, and 1% citrus pectin (cat #, P9135-500G) purchased from Sigma (St. Louis, MO). For this assay 50 ml of medium was added to each 250-ml flask. We evaluated the mycelial growth in a GYEB (1% glucose, 0.5% yeast extract broth) medium and in a minimal medium (0.5% (NH_4_)_2_SO_4_, 0.05% yeast extract, 0.15% KH_2_PO_4_, 0.06% MgSO_4_, 0.06% CaCl_2_, 0.0005% FeSO_4_ 7H_2_O, 0.00016% MnSO_4_ H_2_O, 0.00014% ZnSO_4_ 7H_2_O, and 0.00037% CoCl_2_) supplemented with either 1% glucose or citrus pectin (cat #, P9135-500G, Sigma, St. Louis, MO). Each flask was inoculated with 4–6 x 10^5^ conidia of either *Δpl1332-2* or wild-type *A*. *brassicicola* and incubated in the dark at 25°C with continuous agitation at 100 rpm. The flasks were shaken vigorously by hand several times during the first eight hours to prevent conidia from aggregating and sticking to the wall of the flask. Mycelia were harvested at 4 days postinoculation, washed with distilled water, dried at 70°C overnight, and their dry weights measured.

### Determination of cDNA sequence

Total RNA was extracted from a mixture of plant leaves and wild-type *A*. *brassicicola* at 12 hpi and used to produce cDNA as previously described [36]. Open reading frames of PL1332 and PL4813 were amplified from the cDNA with primer sets PL1332F (TTCACTGCCTTGACCATTACCG) and PL1332 R (CATTGTGCTTTCCGTGGAGT); PL4813F (GGCCAGACTCTGAACATTCC) and PL4813seqR (TTGCATTGCATTCTTTCTCG), respectively. The nucleotide sequence of the PCR products of each gene was determined with the primer sets used for the PCR amplification. Their cDNA sequence was then compared with a known genomic sequence to determine the structure of each gene.

### Quantitative real-time PCR

Expression of the eight pectate lyase genes in wild-type *A*. *brassicicola* was measured by quantitative RT-PCR. We collected mixed samples of fungal and leaf tissue from inoculated *B*. *oleracea* at times that generally represented the five stages of pathogenesis: conidial attachment to the host plant and initiation of germination (4 hpi), penetration (12 hpi), colonization (48 hpi), saprophytic growth on necrotic host tissues (72 hpi), and saprophytic growth and conidiation (120 hpi). Tissues were frozen in liquid nitrogen as soon as they were collected. RNA extraction, cDNA synthesis, and qRT-PCR were performed as previously described [15,34]. Standard curves were produced with purified amplified DNA products of 10 pg/μl, 1 pg/μl, 100 fg/μl, 10 fg/μl, and 1 fg/μl starting concentrations. A baseline subtracted curve fit was used to generate standard curve data. Absolute amounts of transcripts were calculated using correlation coefficient formulae generated from the standard curve in each run with a length correction of 700–800 bp actual transcripts compared to 100–150 bp amplicons. Relative amounts of the transcripts of eight pectate lyase genes were calculated as (transcripts PL / transcripts of *Ef1-α*) x 100. The elongation factor 1-α (*Ef1-α*) was used as a housekeeping gene to normalize transcript amounts of pectate lyase genes because it was the most consistently expressed under all conditions tested based on previous gene expression profile studies during the parasitic and saprophytic growth of wild-type *A*. *brassicicola* [34,36,50,51].

### Expression of PL1332 in *Escherichia coli*

An open reading frame of *PL1332* was amplified from the cDNA with primers PL1332F_BamHI (AAggatccTTCACTGCCTTGACCATTACCG) and PL1332R3_HindIII (CCaagcttCATTGTGCTTTCCGTGGAGT), digested with *Bam*HI and *Hin*dIII and cloned in a pMAL-c2x plasmid (NEB, Ipswich, MA). The plasmid was transformed into *E*. *coli* and selected transformants in the presence of ampicillin. Plasmids purified from 12 selected colonies of transformants were purified and their enzyme digestion patterns examined. Further, the nucleotide sequence of plasmids isolated from three colonies was determined using the M13 forward primer (GTAAAACGACGGCCAGT) to verify the presence of *PL1332* genes and the intact continuous reading frame from the MBP. PL1332 protein produced from this plasmid was translated as a fusion protein from the start codon of maltose binding protein. PL1332 expression by the three transformants was tested and one was selected to produce the enzyme following the protocol in Current Protocols in Molecular Biology (1994), with slight modification. A single colony was transferred from an LB (Luria-Bertani) agar plate to 10 ml of LB broth with ampicillin and incubated overnight at 30°C, and then 1 ml of the cultured inoculum was transferred into 100 ml of LB broth medium. To induce expression of the PL1332 protein, 0.3 mM IPTG was added to the 100-ml culture and incubated for an additional 4–5 hours at 30°C with continuous agitation at 200 rpm. The bacterial cells were harvested and stored at -80°C until use. The stored cells were thawed and resuspended in 30 ml of bind and wash buffer (20 mM Tris-HCl, pH7.4, 200 mM NaCl, 1 mM EDTA, 10 mM β-mercaptoethanol) with one tablet of protease inhibitor (Roche, Basel, Switzeland), 300 μl of phosphatase inhibitor and 1% 4-Nitrophenyl phosphaste di(tri) salt. The cells were disrupted for about 3 minutes in ice-water with 30-second intervals of pulse and pause and 35 amplitudes of an ultrasonic liquid processor (Misonix model: S-4000-010, Newtown, CT). Proteins were further purified with MBP-binding agarose resin following manufacturer’s protocol with slight modification (Elpis Biotech, Daejeon, Korea). A total of 30 ml of supernatant was combined with the prewashed amylose resin and incubated overnight at 4°C for binding. The protein was further washed and eluted with 10 mM maltose in 500 μl bind and wash buffer. We also used a supernatant of whole bacterial lysates for the enzymatic assays instead of purified MBP-PL1332. In addition, the *PL1332* cDNA was cloned in a pGEX-6P1 vector transformed into *E*. *coli*, which produced PL1332-Glutathione S-Transferase (GST) fusion protein. The whole lysate of the *E*. *coli* that produced the PL1332-GST fusion protein was also used for further biological analyses. In these experiments, the supernatant of the whole lysates of the *E*. *coli* transformed with an empty vector was used as a negative control.

### Enzymatic assays

The enzyme activity of pectate lyase was measured by a titrimetric stop reaction method, following a previously described protocol with appropriate modifications [52]. A solution of 5% (w/v) polygalacturonic acid (Cat# P3889, Sigma-Aldrich, St. Louis, MO) at pH 4.0 was mixed with either PL1332 fusion proteins or control proteins to a total volume of 5 ml and incubated at 25°C for 5 minutes. Then 5 ml of 100 mM I_2_ solution and 1 ml of 106 mg/ml Na_2_CO_3_ were added to the reaction mixture and incubated in the dark for 20 minutes. The mixture was then acidified by adding 2 ml of 2.0 N H_2_SO_4_. The free iodine was titrated with continuous stirring against 100 mM Na_2_S_2_O_3_ using 1.0% (w/v) starch as an indicator. We calculated relative amounts of the titrant that measures free iodines that were not covalently bound to oligogalacturonic acids. Enzyme units were calculated using the formula, Units/ml = [(milliliters of titrant for blank- milliliters of titrant for test) x dilution factor x 100] / (0.1 x 5 x 2), and Units/μg protein = (Units/ml enzyme)/(μg protein/ ml enzyme). To visualize the relative amounts of free iodine at the end of the pectate lyase reactions, we added equal amounts of Na_2_S_2_O_3_ to each reaction to sequester the same amount of free iodine. Finally, we added starch to visualize residual iodine.

### Test of necrosis-inducing activity of PL1332 protein

The necrosis-inducing activity of PL1332 was examined with the PL1332 fusion proteins. The proteins were resolved in protein-elution buffer (10 mM maltose, 20 mM Tris-HCl, pH 7.4, 200 mM NaCl, 1 mM EDTA, 10 mM β-mercaptoethanol) or 10 mM Tris-HCl buffer and injected into young leaves of *B*. *oleracea*, *B*. *juncea*, or *B*. *campestris* var. *chinensis* using syringes with 26G x 13 mm needles. The intercellular space became waterlogged with protein solution and the soaked tissue remained visible for several hours. After infiltration, the leaves were maintained in mini humidity chambers and the development of necrotic tissue was observed for up to one week. Infiltration experiments were conducted more than three times and the progress of tissue damage was recorded daily with a digital camera.

## Supporting Information

S1 Fig Alignment of nucleotide sequences between *PL1332* and *PL4813*. Three exons are marked in yellow.
(DOCX)

S2 Fig Enzyme activity of pectate lyase measured by a titrimetric stop reaction method. Enzyme activity was calculated from free iodines that were not oxidized by oligogalacturonic acids, thatoriginated from polygalacturonic acids after digestion by a commercial enzyme (P4716, Sigma-Aldrich, St. Louis, MO) or purified MBP-PL1332 fusion protein. The relative enzyme activity was calculated by relative units ml^-1^ = [(milliliters of titrant for blank- milliliters of titrant for test) x dilution factor x 100] / (0.1 x 2). Units mg^-1^ protein = (Units/ml enzyme)/(mg protein/ ml enzyme). A. Reactions for 1 hour or 16 hours at 25°C. B. Reaction under two different temperatures, 25°C and 37°C. Con: commercial enzyme control, Elu: purified MBP-PL1332 fusion protein.
(EPS)

S3 Fig Necrosis caused by purified MBP-PL1332 fusion proteins on *Brassica juncea* and *B*. *campestris* var. *chinensis*. PL: MBP-PL1332 fusion proteins; MBP: Maltose binding proteins; Buffer: 10 mM maltose in wash/elution buffer (20 mM Tris-HCl, pH7.4, 200mM NaCl, 1mM EDTA, 10mM β-mercaptoethanol); DW: deionized water.
(EPS)

## References

[1] Westman A, Kresovich S, Dickson M (1999) Regional variation in *Brassica nigra* and other weedy crucifers for disease reaction to *Alternaria brassicicola* and *Xanthomonas campestris* pv. *campestris* . Euphytica 106: 253–259.

[2] Sigareva M, Earle E (1999) Camalexin induction in intertribal somatic hybrids between *Camelina sativa* and rapid cycling *Brassica oleracea* . Theor Appl Genet 98: 164–170.

[3] Neergaard P (1945) Danish species of *Alternaria* and *Stemphylium*. London: Oxford University Press.

[4] Warwick SI, Francis A (1994) Guide to the wild germplasm of Brassica and allied crops. Part 5. Life history and geographical data for wild species in the tribe Brassiceae (Cruciferae). Agric Can Res Branch Tech Bull 1994 2E.

[5] Thomma BP, Nelissen I, Eggermont K, Broekaert WF (1999) Deficiency in phytoalexin production causes enhanced susceptibility of *Arabidopsis thaliana* to the fungus *Alternaria brassicicola* . Plant J 19: 163–171. 10476063 10.1046/j.1365-313x.1999.00513.x

[6] Humpherson-Jones FM, Phelps K (1989) Climatic factors influencing spore production in *Alternaria brassicae* and *Alternaria brassicicola* . Annals of Applied Biology 114: 449–458.

[7] Daebeler FD, Riedel A, Riedel V (1986) Wissenschaftliche Zeitschrift der Wilhelm Pieck-Universitat Rostock Naturewissenschaftliche Reiche. 35:52 11636357

[8] Errakhi R, Meimoun P, Lehner A, Vidal G, Briand J, Corbineau F, et al (2008) Anion channel activity is necessary to induce ethylene synthesis and programmed cell death in response to oxalic acid. J Exp Bot 59: 3121–3129. 10.1093/jxb/ern166 18612171

[9] Friesen TL, Stukenbrock EH, Liu Z, Meinhardt S, Ling H, Faris JD, et al (2006) Emergence of a new disease as a result of interspecific virulence gene transfer. Nat Genet 38: 953–956. 16832356 10.1038/ng1839

[10] Kim KS, Min JY, Dickman MB (2008) Oxalic acid is an elicitor of plant programmed cell death during *Sclerotinia sclerotiorum* disease development. Mol Plant Microbe Interact 21: 605–612. 10.1094/MPMI-21-5-0605 18393620

[11] Lehner A, Meimoun P, Errakhi R, Madiona K, Barakate M, Bouteau F (2008) Toxic and signaling effects of oxalic acid: Oxalic acid-Natural born killer or natural born protector? Plant Signal Behav 3: 746–748. 19704845 10.4161/psb.3.9.6634 PMC2634576

[12] Noda J, Brito N, Gonzalez C (2010) The *Botrytis cinerea* xylanase Xyn11A contributes to virulence with its necrotizing activity, not with its catalytic activity. BMC Plant Biol 10: 38 10.1186/1471-2229-10-38 20184750 PMC2844071

[13] Oliver RP, Solomon PS (2008) Recent fungal diseases of crop plants: is lateral gene transfer a common theme? Mol Plant Microbe Interact 21: 287–293. 10.1094/MPMI-21-3-0287 18257678

[14] Pandelova I, Betts MF, Manning VA, Wilhelm LJ, Mockler TC, Ciuffetti LM (2009) Analysis of transcriptome changes induced by *Ptr ToxA* in wheat provides insights into the mechanisms of plant susceptibility. Mol Plant 2: 1067–1083. 10.1093/mp/ssp045 19825681

[15] Cho Y, Davis JW, Kim KH, Wang J, Sun QH, Cramer RA Jr, et al (2006) A high throughput targeted gene disruption method for *Alternaria brassicicola* functional genomics using linear minimal element (LME) constructs. Mol Plant Microbe Interact 19: 7–15. 16404948 10.1094/MPMI-19-0007

[16] Churchill A, LU S, Turgeon B, Yoder O, Macko V (1995) Victorin-deficient REMI mutants of *Cochliobolus victoriae* demonstrate a requirement for victorin in pathogenesis. Fungal Genet Newslett 42A: 41.

[17] Johnson RD, Johnson L, Itoh Y, Kodama M, Otani H, Kohmoto K (2000) Cloning and characterization of a cyclic peptide synthetase gene from *Alternaria alternata* apple pathotype whose product is involved in AM-toxin synthesis and pathogenicity. Mol Plant Microbe Interact 13: 742–753. 10875335 10.1094/MPMI.2000.13.7.742

[18] Yun S-H, Turgeon B, Yoder O (1998) REM-induced mutants of *Mycosphaerella zeae-maydis* lacking the polyketide PM-toxin are deficient in pathogenesis to corn. Physiol Mol Plant Pathol 52: 53–66.

[19] Brandwagt BF, Mesbah LA, Takken FL, Laurent PL, Kneppers TJ, Hille J, et al (2000) A longevity assurance gene homolog of tomato mediates resistance to *Alternaria alternata* f. sp. *lycopersici* toxins and fumonisin B1. Proc Natl Acad Sci U S A 97: 4961–4966. 10781105 10.1073/pnas.97.9.4961 PMC18340

[20] Kohmoto K, Otani H (1991) Host recognition by toxigenic plant pathogens. Experientia 47: 755–764. 1915762 10.1007/BF01922454

[21] Spassieva SD, Markham JE, Hille J (2002) The plant disease resistance gene *Asc-1* prevents disruption of sphingolipid metabolism during AAL-toxin-induced programmed cell death. Plant J 32: 561–572. 12445127 10.1046/j.1365-313x.2002.01444.x

[22] Tanaka A, Shiotani H, Yamamoto M, Tsuge T (1999) Insertional mutagenesis and cloning of the genes required for biosynthesis of the host-specific AK-toxin in the Japanese pear pathotype of *Alternaria alternata* . Mol Plant Microbe Interact 12: 691–702. 10432635 10.1094/MPMI.1999.12.8.691

[23] Tanaka A, Tsuge T (2000) Structural and functional complexity of the genomic region controlling AK-toxin biosynthesis and pathogenicity in the Japanese pear pathotype of *Alternaria alternata* . Mol Plant Microbe Interact 13: 975–986. 10975654 10.1094/MPMI.2000.13.9.975

[24] Ito K, Tanaka T, Hatta R, Yamamoto M, Akimitsu K, Tsuge T, et al (2004) Dissection of the host range of the fungal plant pathogen *Alternaria alternata* by modification of secondary metabolism. Mol Microbiol 52: 399–411. 15066029 10.1111/j.1365-2958.2004.04004.x

[25] Tsuge T, Harimoto Y, Akimitsu K, Ohtani K, Kodama M, Akagi Y, et al (2013) Host-selective toxins produced by the plant pathogenic fungus *Alternaria alternata* . FEMS Microbiol Rev 37: 44–66. 10.1111/j.1574-6976.2012.00350.x 22846083

[26] Wight WD, Kim KH, Lawrence CB, Walton JD (2009) Biosynthesis and role in virulence of the histone deacetylase inhibitor depudecin from *Alternaria brassicicola* . Mol Plant Microbe Interact 22: 1258–1267. 10.1094/MPMI-22-10-1258 19737099

[27] Hashimoto M, Higuchi Y, Takahashi S, Osada H, Sakaki T, Toyomasu T, et al (2009) Functional analyses of cytochrome P450 genes responsible for the early steps of brassicicene C biosynthesis. Bioorg Med Chem Lett 19: 5640–5643. 10.1016/j.bmcl.2009.08.026 19700326

[28] Pedras MS, Chumala PB, Jin W, Islam MS, Hauck DW (2009) The phytopathogenic fungus *Alternaria brassicicola*: phytotoxin production and phytoalexin elicitation. Phytochemistry 70: 394–402. 10.1016/j.phytochem.2009.01.005 19223049

[29] Otani H, Kohnobe A, Kodama M, Kohmoto K (1998) Production of a host-specific toxin by germinating spores of *Alternaria brassicicola* . Physiol Mol Plant Pathol 52: 285–295.

[30] Oka K, Akamatsub H, Kodamab M, Nakajimab H, Kawadab T, Otani H (2005) Host-specific AB-toxin production by germinating spores of *Alternaria brassicicola* is induced by a host-derived oligosaccharide. Physiol Mol Plant P 66: 12–19.

[31] Lawrence CB, Mitchell TK, Craven KD, Cho Y, Cramer RA, Kim KH (2008) Mini review: At Death’s Door: Alternaria Pathogenicity Mechanisms. Plant Pathol J 24: 101–111.

[32] Cho Y (2015) How the necrotrophic fungus *Alternaria brassicicola* kills plant cells remains an enigma Eukaryot Cell In press. 10.1128/EC.00226-14 PMC4385798 25681268

[33] Ohm RA, Feau N, Henrissat B, Schoch CL, Horwitz BA, Barry KW, et al (2012) Diverse lifestyles and strategies of plant pathogenesis encoded in the genomes of eighteen Dothideomycetes fungi. PLoS Pathog 8: e1003037 10.1371/journal.ppat.1003037 23236275 PMC3516569

[34] Srivastava A, Ohm RA, Oxiles L, Brooks F, Lawrence CB, Grigoriev IV, et al (2012) A zinc-finger-family transcription factor, *AbVf19*, is required for the induction of a gene subset important for virulence in *Alternaria brassicicola* . Mol Plant Microbe Interact 25: 443–452. 10.1094/MPMI-10-11-0275 22185468

[35] Tonukari NJ, Scott-Craig JS, Walton JD (2000) The *Cochliobolus carbonum* SNF1 gene is required for cell wall-degrading enzyme expression and virulence on maize. Plant Cell 12: 237–248. 10662860 10.1105/tpc.12.2.237 PMC139761

[36] Cho Y, Ohm RA, Grigoriev IV, Srivastava A (2013) Fungal-specific transcription factor *AbPf2* activates pathogenicity in *Alternaria brassicicola* . Plant J 75: 498–514. 10.1111/tpj.12217 23617599

[37] Cosgrove DJ (2005) Growth of the plant cell wall. Nat Rev Mol Cell Biol 6: 850–861. 16261190 10.1038/nrm1746

[38] Willats WG, McCartney L, Mackie W, Knox JP (2001) Pectin: cell biology and prospects for functional analysis. Plant Mol Biol 47: 9–27. 11554482

[39] Caffall KH, Mohnen D (2009) The structure, function, and biosynthesis of plant cell wall pectic polysaccharides. Carbohydr Res 344: 1879–1900. 10.1016/j.carres.2009.05.021 19616198

[40] Oeser B, Heidrich PM, Muller U, Tudzynski P, Tenberge KB (2002) Polygalacturonase is a pathogenicity factor in the *Claviceps purpurea*/rye interaction. Fungal Genet Biol 36: 176–186. 12135573 10.1016/s1087-1845(02)00020-8

[41] Shieh MT, Brown RL, Whitehead MP, Cary JW, Cotty PJ, Cleveland TE, et al (1997) Molecular genetic evidence for the involvement of a specific polygalacturonase, P2c, in the invasion and spread of *Aspergillus flavus* in cotton bolls. Appl Environ Microbiol 63: 3548–3552. 9293005 10.1128/aem.63.9.3548-3552.1997 PMC168660

[42] ten Have A, Mulder W, Visser J, van Kan JA (1998) The endopolygalacturonase gene *Bcpg1* is required for full virulence of *Botrytis cinerea* . Mol Plant Microbe Interact 11: 1009–1016. 9768518 10.1094/MPMI.1998.11.10.1009

[43] Davis KR, Lyon GD, Darvill AG, Albersheim P (1984) Host-pathogen interactions: XXV. Endopolygalacturonic acid lyase from *Erwinia carotovora e*licits phytoalexin accumulation by releasing plant cell wall fragments. Plant Physiol 74: 52–60. 16663385 10.1104/pp.74.1.52 PMC1066623

[44] Djamei A, Schipper K, Rabe F, Ghosh A, Vincon V, Kahnt J, et al (2011) Metabolic priming by a secreted fungal effector. Nature 478: 395–398. 10.1038/nature10454 21976020

[45] Doehlemann G, van der Linde K, Assmann D, Schwammbach D, Hof A, Mohanty A, et al (2009) Pep1, a secreted effector protein of *Ustilago maydis*, is required for successful invasion of plant cells. PLoS Pathog 5: e1000290 10.1371/journal.ppat.1000290 19197359 PMC2631132

[46] Ciuffetti LM, Manning VA, Pandelova I, Betts MF, Martinez JP (2010) Host-selective toxins, Ptr ToxA and Ptr ToxB, as necrotrophic effectors in the *Pyrenophora tritici-repentis*–wheat interaction. New Phytol 187: 911–919. 10.1111/j.1469-8137.2010.03362.x 20646221

[47] Kale SD, Gu B, Capelluto DG, Dou D, Feldman E, Rumore A, et al (2010) External lipid PI3P mediates entry of eukaryotic pathogen effectors into plant and animal host cells. Cell 142: 284–295. 10.1016/j.cell.2010.06.008 20655469

[48] Kale SD, Tyler BM (2011) Entry of oomycete and fungal effectors into plant and animal host cells. Cell Microbiol 13: 1839–1848. 10.1111/j.1462-5822.2011.01659.x 21819515

[49] Cho Y, Kim K-H, La Rota M, Scott D, Santopietro G, Callihan M, et al (2009) Identification of virulence factors by high throughput targeted gene deletion of regulatory genes in *Alternaria brassicicola* . Mol Microbiol 72: 1316–1333. 10.1111/j.1365-2958.2009.06689.x 19460100

[50] Cho Y, Srivastava A, Ohm RA, Lawrence CB, Wang KH, Grigoriev IV, et al (2012) Transcription factor *Amr1* induces melanin biosynthesis and suppresses virulence in *Alternaria brassicicola* . PLoS Pathog 8: e1002974 10.1371/journal.ppat.1002974 23133370 PMC3486909

[51] Cho Y, Ohm RA, Devappa R, Lee HB, Grigoriev IV, Kim BY, et al (2014) Transcriptional Responses of the *Bdtf1*-Deletion Mutant to the Phytoalexin Brassinin in the Necrotrophic Fungus *Alternaria brassicicola* . Molecules 19: 10717–10732. 10.3390/molecules190810717 25061722 PMC6270968

[52] Starr MP, Chatterjee AK, Starr PB, Buchanan GE (1977) Enzymatic degradation of polygalacturonic acid by Yersinia and Klebsiella species in relation to clinical laboratory procedures. J Clin Microbiol 6: 379–386. 334794 10.1128/jcm.6.4.379-386.1977 PMC274778

