# Supplementary material for: A Pectate Lyase-Coding Gene Abundantly Expressed during Early Stages of Infection Is Required for Full Virulence in Alternaria brassicicola
Source: PLoS One. 2015 May 21;10(5):e0127140. doi: 10.1371/journal.pone.0127140 (PMC4440746; doi:10.1371/journal.pone.0127140)
Supplement: S1 Fig — Three exons are marked in yellow. (DOCX) [file pone.0127140.s001.docx]

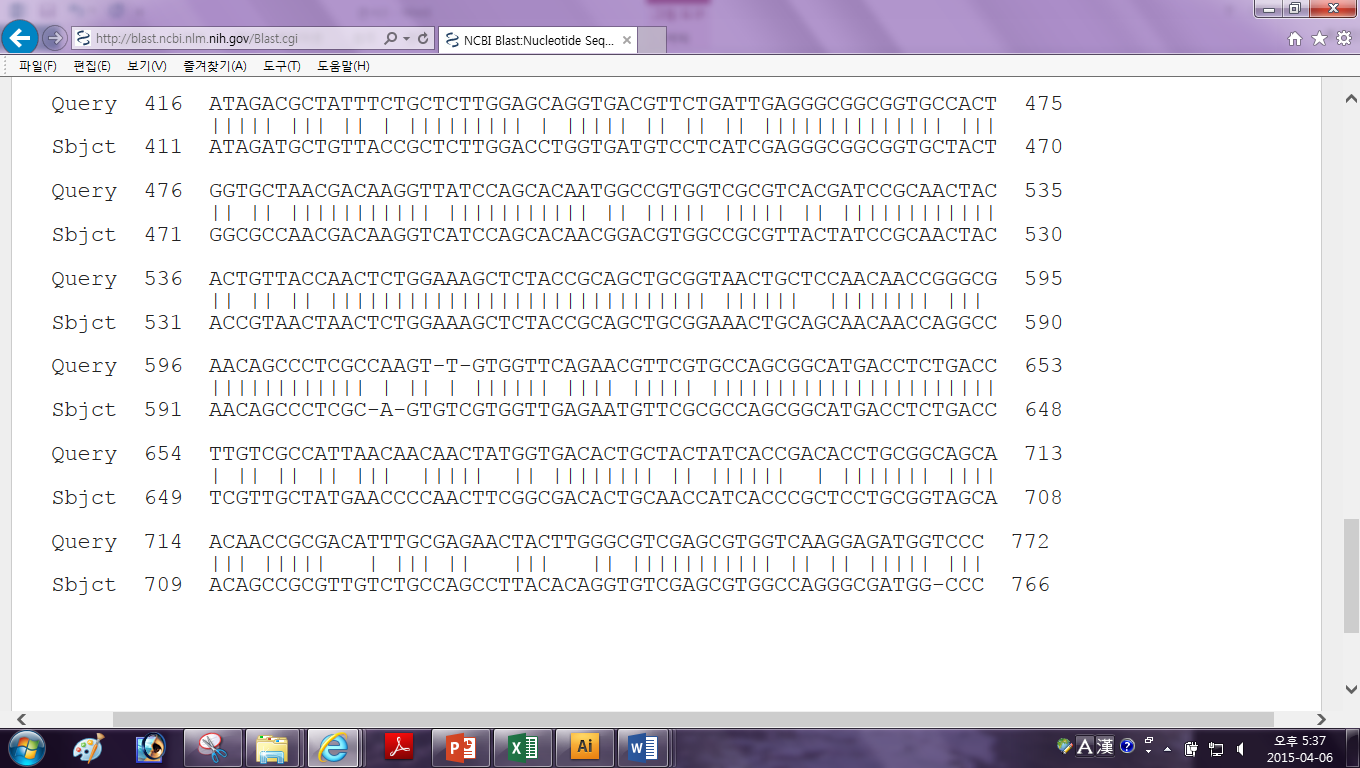

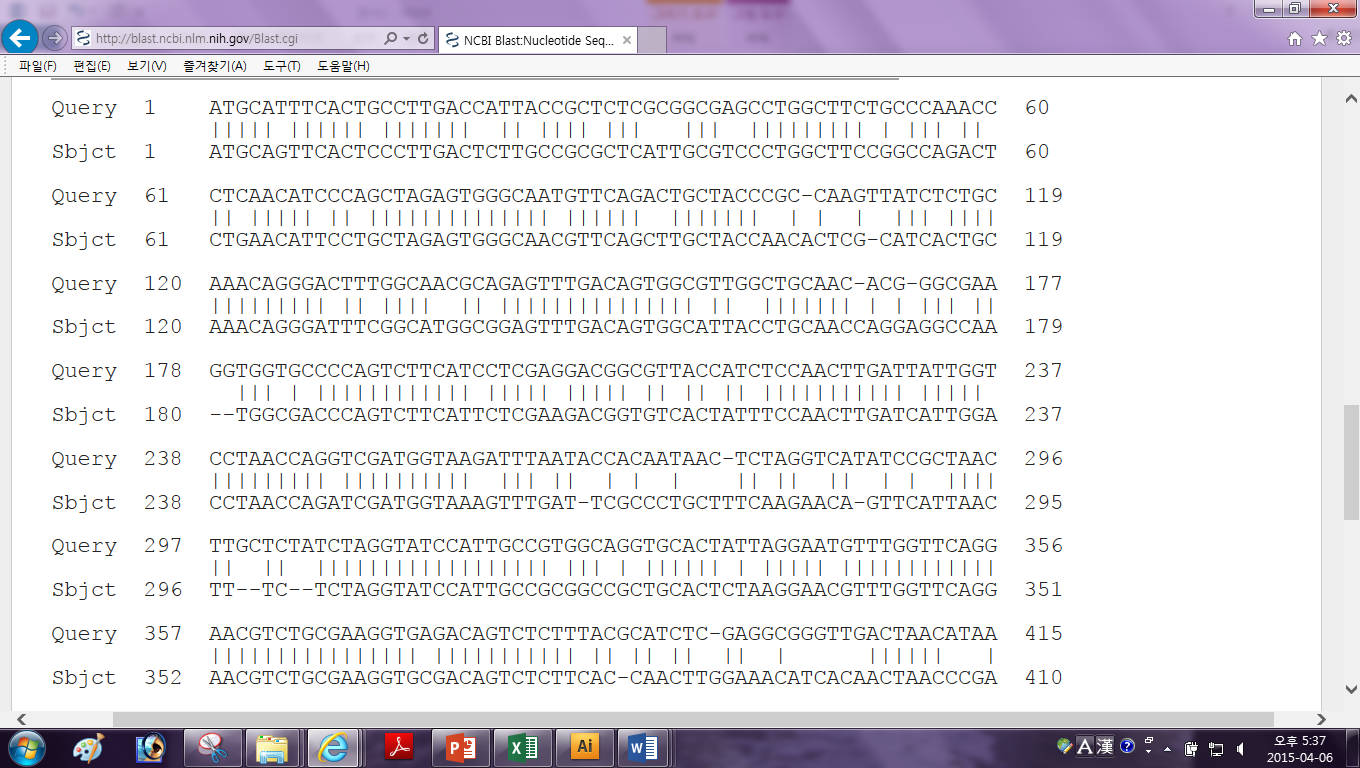


PL1332

PL4813

PL1332

PL4813

PL1332

PL4813

PL1332

PL4813

PL1332

PL4813

PL1332

PL4813

PL1332

PL4813

PL1332

PL4813

PL1332

PL4813

PL1332

PL4813

PL1332

PL4813

PL1332

PL4813

PL1332

PL4813

**S1 Fig. Alignment of nucleotide sequences between *PL1332* and *PL4813***.
